# Bilateral cerebellar high-frequency rTMS for refractory epilepsy: a randomized, single-blind, sham-controlled study

**DOI:** 10.3389/fneur.2026.1952421

**Published:** 2026-10-29

**Authors:** Anru Liu, Lin Li, Yuxiang Han, Lijun Wan, Juntao Zhang, Kejun Zang, Xuewu Liu

**Affiliations:** 1 Department of Neurology, Shandong Provincial Hospital, Shandong University, Jinan, China; 2 Department of Neurology, Qilu Hospital, Shandong University, Jinan, China; 3 Department of Neurology, Shandong Provincial Hospital Affiliated to Shandong First Medical University, Jinan, China; 4 Department of Neurology, The First Affiliated Hospital of Shandong First Medical University & Shandong Provincial Qianfoshan Hospital, Shandong Institute of Neuroimmunology, Shandong Key Laboratory of Rheumatic Disease and Translational Medicine, Jinan, China

**Keywords:** biomarker, cerebellum, clinical outcome, electrophysiology, neuromodulation, non-invasive brain stimulation

## Abstract

**Background:**

For patients with drug-resistant epilepsy who are not suitable for surgery, transcranial magnetic stimulation (TMS) is an important non-invasive neuromodulation therapy. The cerebellar cortex can inhibit cortical excitability via the cerebello-thalamo-cortical pathway. Here, we explore whether high-frequency (20 Hz) cerebellar repetitive TMS (rTMS) reduces seizure frequency and interictal epileptiform discharges (IEDs) in refractory epilepsy.

**Methods:**

Eighty-seven patients with refractory epilepsy were included in the intention-to-treat (ITT) population (*n* = 87) and then randomized to active rTMS (*n* = 44) or sham stimulation (*n* = 43). One patient in treatment group had no post-baseline efficacy data; efficacy analyses were therefore performed in the modified intention-to-treat (mITT) population (*n* = 86). The active group received two cycles of 20 Hz rTMS targeting the cerebellum. Seizure frequency and IEDs were assessed before and after treatment.

**Results:**

Poisson generalized linear mixed models revealed a significant group×time interaction for seizure frequency [*F*(2,236) = 162.65, *p* < 0.001]. The active group had significantly lower seizure frequencies at months 1, 2, and 3 (all *p* < 0.001), with the largest difference at month 2. Percentage reductions of seizure frequency in the active group were 64.6%, 73.2%, and 13.3%, respectively, compared with 3.9%, 0.6%, and −8.4% in the control group (all *P* < 0.001). Responder rates (≥50% reduction) in the active group were 90.7%, 95.3%, and 0% vs. 0% in controls (*p* < 0.001 for months 1–2). Within-group analysis showed significant IED reduction after active rTMS (*p* < 0.001) but no change in controls. Moreover, a significant group × baseline interaction (*p* < 0.001) indicated greater suppression in patients with higher baseline IEDs. Exploratory subgroup analysis further confirmed this. All adverse events were mild, with no significant difference in the incidence between the two groups.

**Conclusion:**

Active cerebellar rTMS was associated with reduced seizure frequency, improved responder rates, and suppressed IEDs in refractory epilepsy with no obvious discomfort. The optimal efficacy duration was approximately 2 months, with greater IED suppression in patients with higher baseline levels.

## 1 Introduction

Antiseizure medications (ASMs), including levetiracetam, valproate, carbamazepine, lamotrigine, and oxcarbazepine, remain the first-line treatment for epilepsy, but about 30% of patients are drug resistant (1). The International League Against Epilepsy defines DRE as failure of adequate trials of two tolerated, appropriately chosen and used ASM schedules, whether as monotherapies or in combination, to achieve sustained seizure freedom (2). For drug-resistant epilepsy (DRE), resective or disconnective surgery is limited to patients with a well-localized epileptogenic zone that can be safely resected or disconnected, such as in glioma-related epilepsy (3). These procedures are irreversible and may cause memory, language, motor, and visual deficits, as well as an increased risk of infection (4).

Neuromodulation has become an important treatment strategy for DRE. Transcranial magnetic stimulation (TMS) is a non-invasive neuromodulation method. TMS uses a rapidly changing magnetic field to induce an electric field in superficial neural tissue, depolarizing susceptible axons and recruiting local and connected neural circuits; repeated stimulation can produce activity-dependent changes in network excitability that outlast the stimulation train. It does not require electrode implantation, avoids direct electrode-tissue contact, and avoids charge transfer and electrode corrosion (5). Repetitive transcranial magnetic stimulation (rTMS) has level A or B evidence in several neurological and psychiatric disorders, such as major depressive disorder, chronic neuropathic pain, post-acute motor recovery after stroke, fibromyalgia, post-traumatic stress disorder, and selected motor or depressive symptoms of Parkinson’s disease (6). In epilepsy, TMS is used for diagnosis, research, and treatment (4). Previous studies showed that high-frequency rTMS (HF-rTMS; >5 Hz) usually increases cortical excitability, whereas low-frequency rTMS (LF-rTMS; ≦1 Hz) has inhibitory effects (7, 8). This conventional frequency rule is derived mainly from direct motor-cortex stimulation and motor-evoked-potential measurements. Clinical studies showed that LF-rTMS can reduce seizure frequency in patients with DRE (9, 10). These findings suggest that rTMS may provide a reversible and non-invasive treatment option for patients who are not candidates for surgery.

In addition to clinically visible seizure frequency, IED frequency is also an important indicator for evaluating the efficacy of epilepsy treatment. Interictal epileptiform discharges (IEDs) are brief, abnormal, highly synchronized cortical electrical activities that occur during the interictal period in patients with epilepsy. Clinically, they typically manifest as spikes, sharp waves, polyspikes, or spike-and-wave complexes, and are not accompanied by observable clinical seizure symptoms. The International Federation of Clinical Neurophysiology (IFCN) proposed an operational definition of IEDs in 2017 (11), and its clinical diagnostic performance was validated by Kural et al. in 2020 (12). A series of studies have investigated the role of TMS in reducing IED frequency. Low-frequency rTMS at 0.5 Hz and 90% rMT, delivered at high intensity targeting the epileptogenic zone, can simultaneously reduce seizure frequency and IEDs, with efficacy lasting up to 8 weeks (10). She et al. found that active rTMS reduced IED frequency in patients with SeLECTS (self-limited epilepsy with centrotemporal spikes) and significantly reduced pathological beta-band hyperconnectivity between multiple brain regions (13). Other studies have reported that low-frequency rTMS targeting the epileptogenic zone in neocortical focal epilepsy only produced a trend toward reduced IEDs, despite a significant reduction in clinical seizures (14). These findings collectively indicate that rTMS can exert definite antiepileptic effects on both clinical seizures and electrophysiological phenotypes. The scientific community is currently exploring a shift in the role of IEDs in neuromodulation therapy; researchers are no longer limited to using IEDs solely as an outcome measure of treatment efficacy, but are exploring their potential as trigger signals for closed-loop TMS stimulation. Makkonen et al. have preliminarily realized this concept using a real-time EEG-CNN (convolutional neural network) algorithm; however, the actual hit rate of TMS pulses within the IED window is currently only 19–39%, leaving room for improvement (15).

However, the optimal stimulation target for different epilepsy types remains unclear. The target may not be limited to the epileptogenic zone and may depend on anatomical connectivity, downstream discharge modulation, and accessibility (16). The cerebello-thalamo-cortical pathway is involved in essential tremor, Parkinson’s disease, Wilson’s disease, multiple system atrophy, and schizophrenia-spectrum disorders (17–22). Reduced inhibitory influence of this pathway on the primary motor cortex has been linked to familial cortical myoclonic tremor with epilepsy (23–25). Since the use of deep brain stimulation (DBS), the cerebellar cortex has been explored as a treatment target for epilepsy (26). Purkinje cells, the output neurons of the cerebellar cortex, send inhibitory GABAergic projections to the deep cerebellar nuclei (DCN). The largest DCN, dentate nucleus (DN), further influences multiple cortical areas through the thalamus (27). Therefore, cerebellar cortical stimulation may reduce cortical excitability by modulating the cerebello-thalamo-cortical network.

Prior epilepsy studies have used both 5-Hz cerebellar cortical stimulation and inhibitory continuous theta-burst stimulation of the dentate region (28, 29). Given that Purkinje fibers in the cerebellar cortex exert inhibitory effects on the downstream dentate nucleus, both studies theoretically achieve cortical-inhibitory effects via suppression of the cerebellar dentate nucleus. Based on this rationale, we used HF-rTMS (20 Hz) to stimulate the cerebellar cortex. High-frequency cerebellar stimulation may recruit Purkinje cells and thereby increase inhibitory control over the DCN, potentially reducing downstream thalamocortical drive (27, 30–32). But strictly speaking, the usual low-frequency/inhibitory and high-frequency/facilitatory rule cannot be assumed to transfer unchanged from direct motor-cortex stimulation to a remote network target such as the cerebellum. Actually, we had no preliminary physiological data or computational model demonstrating that this protocol produces net cortical inhibition in DRE; this mechanism is therefore a testable hypothesis rather than an established explanation. Future basic experimental studies are required to verify whether excitation of cerebellar Purkinje cells can produce cortical inhibition and reduce epileptic seizures via the cerebello-thalamo-cortical circuit.

In this study, The primary objective was to assess the efficacy and duration of cerebellar HF-rTMS in reducing seizure frequency and interictal epileptiform discharges in patients with DRE (benefit). The secondary objective was to monitor the incidence of adverse events (harm).

## 2 Patients and methods

### 2.1 Patients

We enrolled 87 patients between November 2018 and September 2021, who had been diagnosed with refractory epilepsy at Qilu Hospital of Shandong University. All patients met the following inclusion criteria: (1) According to the classification for epileptic seizures published by the International League Against Epilepsy in 2017 (33), patients were attacked by focal seizures, generalized seizures, or focal to bilateral tonic–clonic seizures; (2) All patients met the standards of drug-resistant epilepsy according to ILAE, which is defined as failure of adequate trials of two tolerated, appropriately chosen and used antiepileptic drug schedules (whether as monotherapies or in combination) to achieve sustained seizure freedom (2); (3) The frequency of epileptic seizures (various types, Table 1) was more than 4 times per month; (4) The daily dose of antiepileptic drugs was unchanged during 3 months before treatment and 3 months after treatment, and there were no serious side effects (serious drug eruptions, etc.); (5) The history of illness was at least 2 years. Exclusion criteria were as follows: (1) progressive, evolving brain pathologies (e.g., progressive brain tumor, Rasmussen encephalitis, continuously worsening neurodegenerative disease) or an unstable systemic disease that could interfere with participation or outcome assessment; (2) history of alcohol abuse; (3) history of status epilepticus, psychotic seizures, or history of heart, lung, and renal insufficiency; (4) patients with contraindications to rTMS: implanted with cardiac pacemakers, vagus nerve stimulators, or other metallic implants or with elevated intracranial pressure; (5) inability to cooperate with treatment or complete reliable outcome assessment. Patients who met any of the exclusion criteria were excluded from enrollment.

**Table 1:** Baseline patient characteristics in the ITT set.

Patient characteristics	Treatment group (*n* = 44)	Control group (*n* = 43)	Statistic	P (two-sided test)
Age, year, median (IQR)^a^	22.30 (14.60–28.38)	20.50 (16.70–25.50)	*Z* = −0.085	0.932
Sex, male/female, *n* (%)^b^	22 (50.0)/ 22 (50.0)	25 (58.1)/ 18 (41.9)	*χ*^2^ = 0.580	0.446
Disease duration, year, median (IQR)^a^	11.20 (6.70–15.00)	10.40 (7.50–13.40)	*Z* = −0.302	0.763
Baseline seizure frequency/month, $\bar{x}$ ±SD^c^	6.55 ± 1.17	6.43 ± 0.81	*t* = 0.514	0.608
Seizure type, *n* (%)^d^ GTCS FICS/GTCS FBTCS Tonic/GTCS FPCS/FBTCS Absence/FBTCS FICS/tonic/GTCS Absence/tonic/GTCS Absence/tonic/ FBTCS	8 (18.2) 6 (13.6) 24 (54.5) 1 (2.3) 1 (2.3) 1 (2.3) 0 (0.0) 2 (4.5) 1 (2.3)	13 (30.2) 2 (4.7) 22 (51.2) 1 (2.3) 1 (2.3) 0 (0.0) 1 (2.3) 2 (4.7) 1 (2.3)	—	0.761
Epilepsy Type, *n* (%)^b^ Focal Generalized Combined	25 (56.8) 11 (25.0) 8 (18.2)	23 (53.5) 16 (37.2) 4 (9.3)	*χ*^2^ = 2.331	0.312
Etiology, *n* (%)^d^ Structural Genetic Immune Infectious Unknown	12 (27.3) 2 (4.5) 5 (11.4) 2 (4.5) 23 (52.3)	11 (25.6) 2 (4.7) 6 (14.0) 3 (7.0) 21 (48.8)	—	0.982
MRI findings, *n* (%)^b^ Lesional Non-lesional	26 (59.1) 18 (40.9)	24 (55.8) 19 (44.2)	*χ*^2^ = 0.096	0.757
Combined ASMs, *n* (%)^d^ Two types Three types Four types	15 (34.1) 26 (59.1) 3 (6.8)	16 (37.2) 24 (55.8) 3 (7.0)	—	0.942

^a^ Mann–Whitney U test; ^b^ Pearson’s chi-square test; ^c^ independent-samples T test; ^d^ Fisher’s exact test.

FPCS: focal preserved consciousness seizure; FICS: focal impaired consciousness seizure; FBTCS: focal to bilateral tonic–clonic seizures; GTCS: generalized tonic–clonic seizures; ASMs, antiseizure medications; MRI, magnetic resonance imaging; IQR, interquartile range; SD, standard deviation.

This study was conducted in accordance with the Declaration of Helsinki and approved by the ethics committee of Qilu Hospital of Shandong University, with the approval number: [NO. KYLL-2018-367]. All patients provided informed consent to participate in the study and were enrolled after ethics approval. However, this study was conducted and completed prior to the implementation of the current clinical trial registration policy at our institution. Due to the lengthy interval since trial completion, the institutional ethics committee was unable to provide the updated continuing review documentation required for retrospective registration. Consequently, this trial could not be retrospectively registered. We acknowledge that the absence of prospective/retrospective trial registration limits the generalizability and interpretability of our findings, as registration is essential for ensuring transparency and reducing reporting bias. Nevertheless, all outcome assessments were performed under strict blinded conditions, data collection and analysis were completed prospectively according to a prespecified protocol, and the results are reported in full accordance with the CONSORT guidelines where applicable. Future studies should ensure that trial protocols are registered prior to patient enrollment and that all regulatory documentation is maintained to enhance the reliability and external validity of the evidence.

Patients and the public were not formally involved in the trial design or conduct. However, prior to finalizing the study protocol, the research team informally consulted five patients and two family caregivers regarding the feasibility of the intervention schedule (two cycles of daily sessions) and the burden of follow-up visits. Their feedback led to the provision of flexible appointment times and reimbursement for travel costs. Patients were not involved in the analysis, interpretation, or writing of the manuscript. No important changes were made to the trial protocol after commencement.

### 2.2 Study design

The study was a randomized, single-blind, parallel-controlled trial. The enrolled patients were randomly divided into two groups: 44 in the treatment group and 43 in the control group (the two groups were paired in seizure type and frequency, as shown in Table 1). These 87 randomized patients were retained for allocation reporting and baseline accounting, constituting the ITT population. During the study, one patient in the treatment group was lost to follow-up before any post-baseline efficacy assessment. Consequently, 86 patients received the allocated intervention and had evaluable post-baseline data, with 43 in each group. These 86 patients comprised the mITT population used for all efficacy analyses. The participant flow is shown in Figure 1.

**Figure 1:**
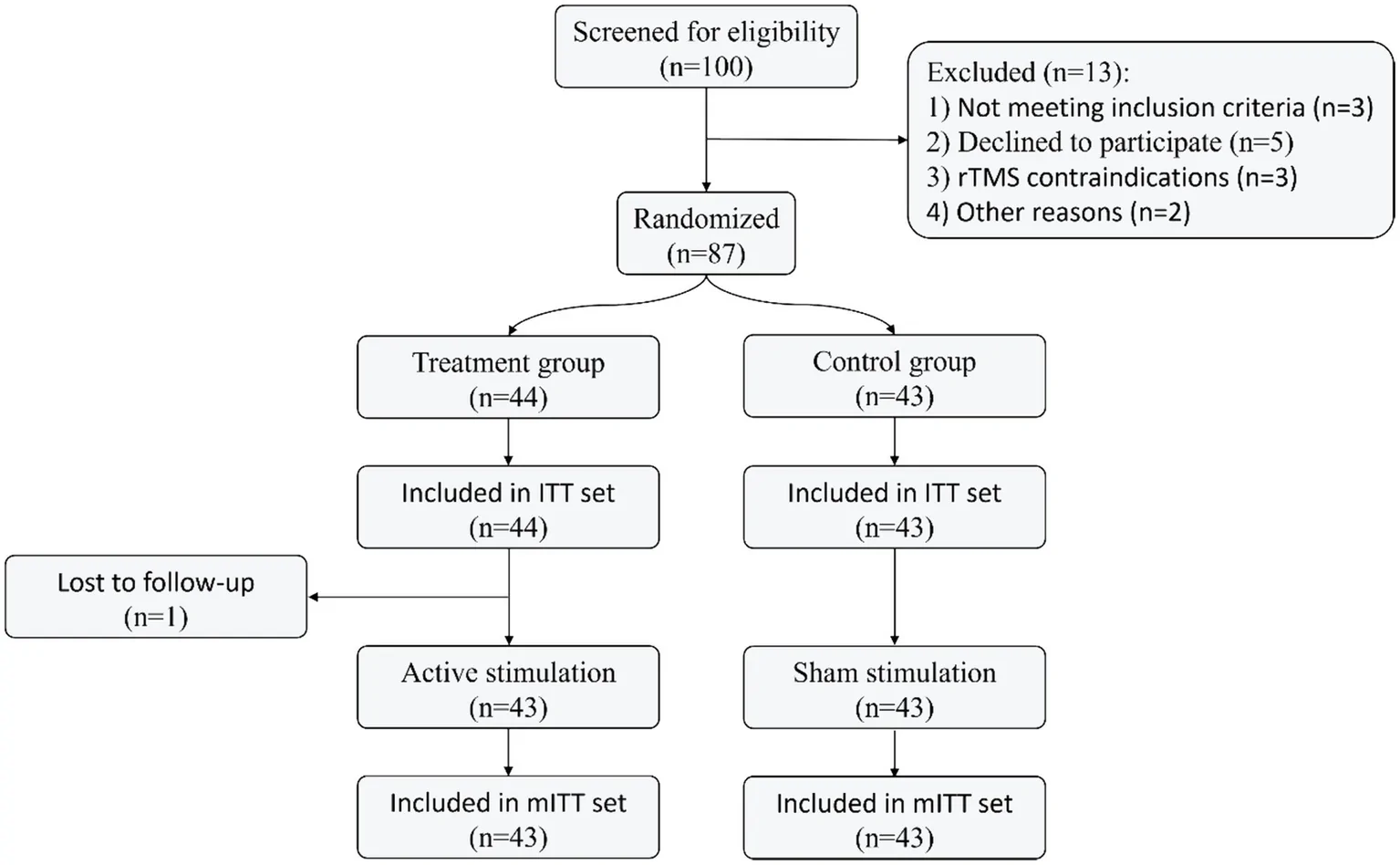
Participant flow diagram. Not meeting inclusion criteria (*n* = 3): two had baseline seizure frequency less than 4 seizures per month, and one had a disease duration of less than 2 years.

An independent coordinator performed stratified block randomization, with stratification by sex. Within each stratum, randomly mixed block sizes of 4 or 6 were used, and the randomization sequence for each block was generated using SPSS software, ensuring equal group sizes within each block. The resulting group assignments were sequentially numbered, placed in sealed, opaque envelopes, and subsequently opened in order by another physician responsible for patient enrollment. The treating physician was not blinded. Although the sham stimulation was sensorially distinguishable, patients were intended to be blinded, and outcome assessors, EEG readers, and statisticians were blinded to group allocation.

The rTMS intervention was delivered by neurologists with at least 3 years of clinical experience who had completed standardized rTMS training. In this study, we use the Magstim Rapid2 magnetic stimulator (made in the UK) and the figure-eight coil. The target was laid 2 cm below and 2 cm aside the top of the inion. The same fixed landmark procedure was used for all participants; MRI-guided neuronavigation, subject-specific anatomical adjustment, and electric-field modeling were not used. The center of the figure-eight coil was aligned with the stimulus target, and the plane of the coil was parallel to the skull in the treatment group, the latter stuck closely to the scalp. Sham stimulation was delivered using identical parameters, except that the stimulating coil was tilted 90° on one wing, with only its edge in contact with the scalp. Although this configuration cannot perfectly replicate the scalp sensations and acoustic effects of active stimulation, it can still induce partial paresthesia in patients, without eliciting substantial cerebral effects.

The resting motor threshold (rMT) was defined as the minimal TMS stimulation intensity applied to the scalp that was sufficient to elicit observable movements in the right hand. Patients in the treatment group, while maintaining their conventional antiepileptic drug regimens, underwent high-frequency (20 Hz), high-intensity (90% rMT) rTMS targeting the bilateral cerebellum. The treatment consisted of 1,200 stimuli per day, administered over five consecutive days to form one treatment cycle. A total of two continuous cycles were completed. The control group underwent sham stimulation using the same protocol. To mitigate potential hearing damage from the device’s operation, all patients were provided with earplugs.

Following completion of the main trial randomization and before the start of treatment, 22 patients were randomly sampled from each group, using a random number table with stratification by sex, to undergo 48-h long-range video EEG (VEEG) monitoring. No additional restrictions based on demographic or clinical features were applied. All patients selected for VEEG recording provided informed consent prior to enrollment. Eligibility for the VEEG subgroup required that patients: had been randomized in the main trial; consented to 48-h VEEG monitoring; were able to attend both pre and post-treatment VEEG sessions within the scheduled time frame; and had interpretable VEEG recordings suitable for IED quantification. Within one week before and after the treatment, these patients underwent 48-h long-range video EEG (VEEG) monitoring. The recordings were acquired using a 32-channel Nicolet EEG system with scalp electrodes positioned according to the international 10–20 system (34). All analyzable scalp channels were reviewed; no single channel or cerebral lobe was pre-specified because IED topography varied among patients. Epochs or channels with obvious ocular, muscle, movement, electrode, or other non-cerebral artifact were not used for visual counting. Because recordings were obtained outside stimulation sessions, contemporaneous stimulation artifacts were not present. Interictal epileptiform discharges (IEDs) were identified by clinical visual review as spikes, sharp waves, spike-and-wave complexes, or polyspike-and-wave complexes with a plausible cerebral field and morphology distinct from background activity (12). The recordings were evaluated by an electrophysiologist and a neurologist. Separate reader scores were not retained, so formal inter-rater reliability could not be calculated. Recordings were excluded when persistent artifact or poor signal quality prevented reliable IED quantification; the archived protocol did not specify a numerical exclusion threshold. Exact acquisition and filter settings could not be reconstructed reliably from the archived material. After excluding 3 cases in the treatment group and 1 case in the control group due to poor EEG recording quality, 19 patients in the treatment group and 21 patients in the control group were finally included in the VEEG subgroup for statistical analysis.

### 2.3 Assessments

The experimental protocol is outlined in Figure 2. Seizure frequency during the 3 months preceding treatment was designated as the baseline. Follow-up assessments were conducted over the 3 months following treatment to document seizure occurrence. Patients or family caregivers recorded events prospectively on paper seizure diary cards. At each follow-up, two neurology clinicians reviewed the entries together with the patient’s or caregiver’s description and available clinical records; only events judged clinically compatible with the patient’s habitual seizures were included. VEEG confirmation was not required for every outpatient event. Because seizure ascertainment depended on patient or caregiver recording, unwitnessed or unrecorded seizures may have been missed. Caregiver support and diary completeness were not measured systematically, so differential under-reporting among less-supported patients cannot be excluded. VEEG recordings were evaluated by both an electrophysiologist and a neurologist. Regarding epileptiform discharges, the frequency of discharges per minute, as recorded by the 48-h VEEG performed before treatment, served as the baseline and was compared against the post-treatment recording. A representative ictal VEEG recording is shown in Figure 3.

**Figure 2:**
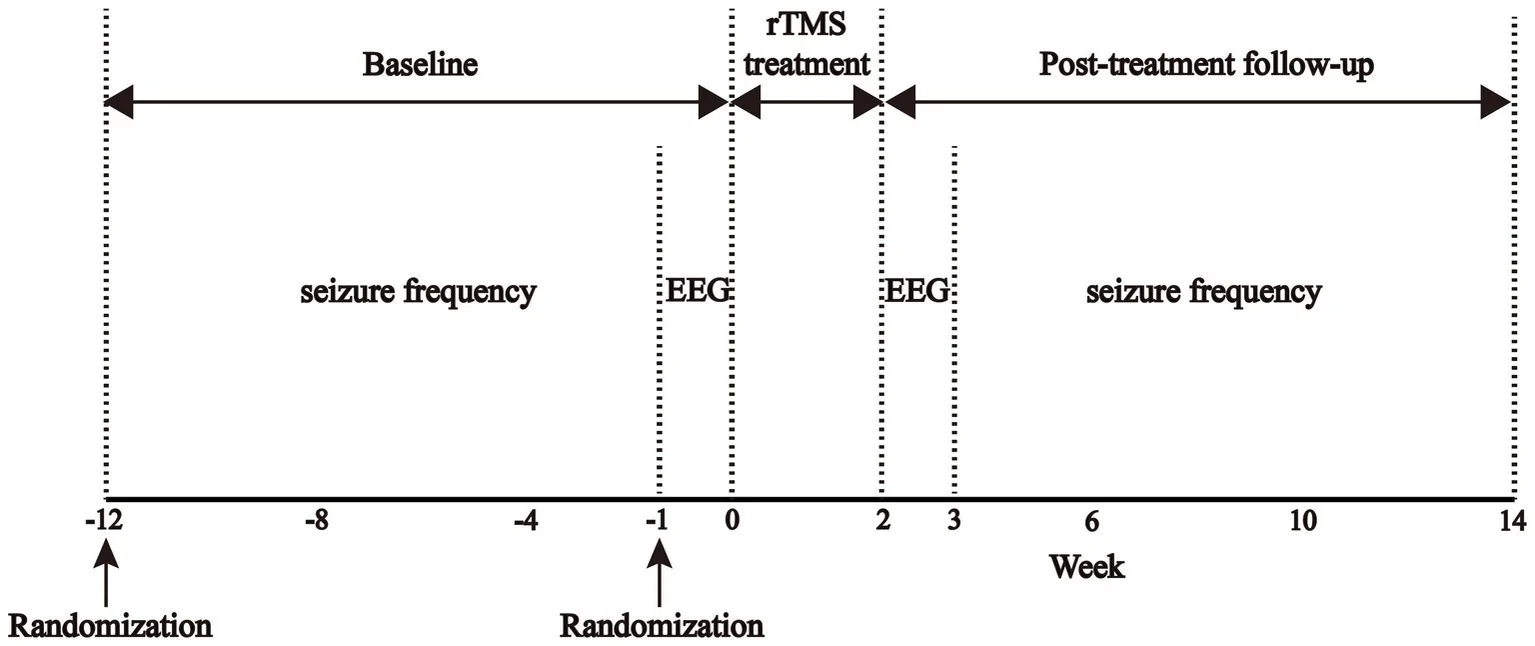
Experimental procedure.

**Figure 3:**
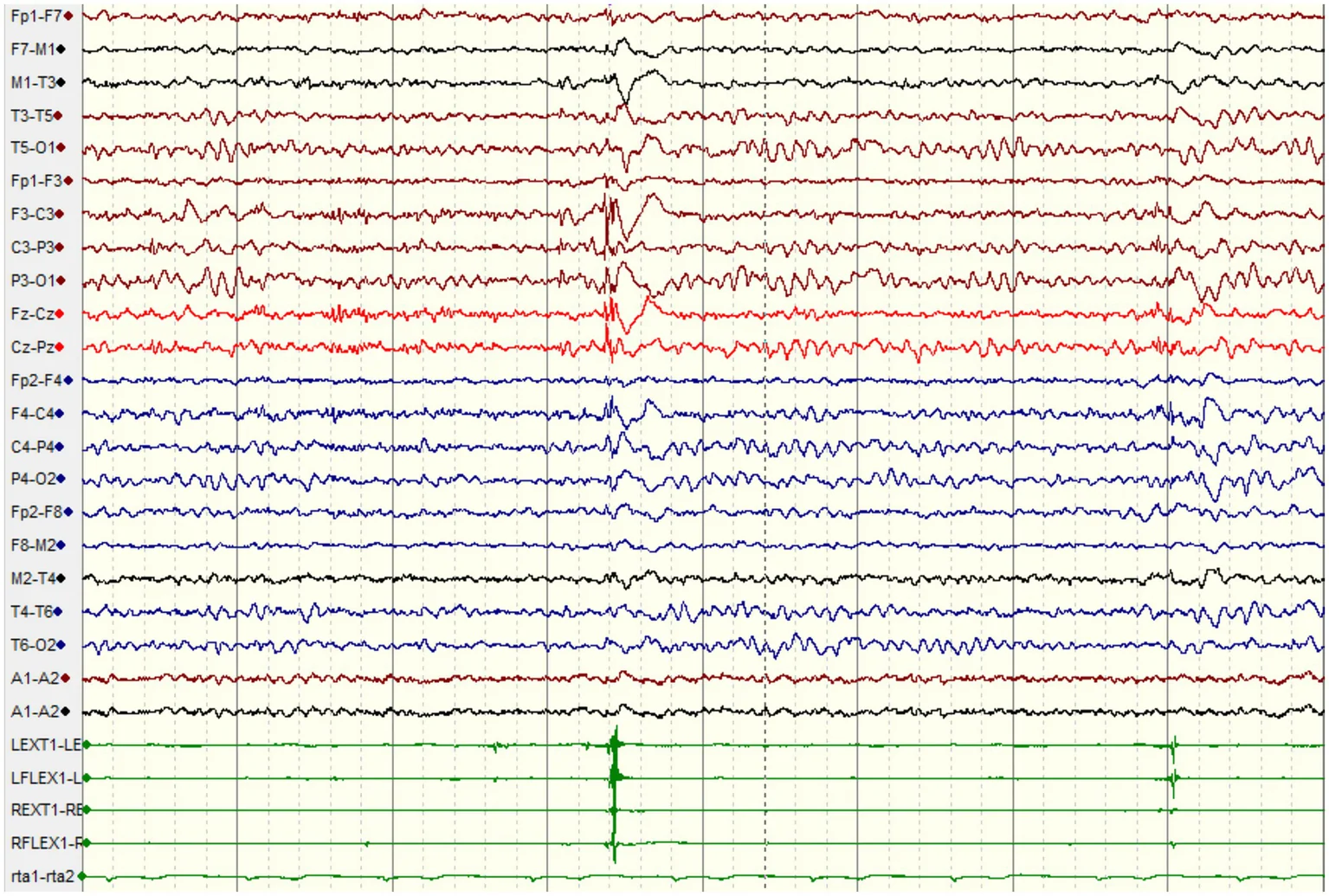
Ictal VEEG revealed spikes, polyspike-and-wave complexes, and synchronous electromyographic (EMG) changes.

### 2.4 Statistical analysis

Efficacy analyses were performed in the modified intention-to-treat (mITT) population, defined as randomized patients who received the allocated intervention and had at least one post-baseline efficacy assessment. The primary outcome was monthly seizure frequency during follow-up. Because this outcome was repeated-measure count data with a skewed distribution and was affected by baseline seizure frequency, it was analyzed with a generalized linear mixed model (GLMM). The variance-to-mean ratios of follow-up seizure-frequency data indicated underdispersion rather than overdispersion. In addition, the dispersion parameter estimated from the negative binomial GLMM was approximately zero (3.049 × 10–9), suggesting that the negative binomial model effectively collapsed to a Poisson form. Therefore, a Poisson-distribution GLMM with a log link was selected.

The percentage reduction in seizure frequency was analyzed with a linear mixed model (LMM). To assess multicollinearity, all fixed-effect variables of LMM model were entered as independent variables into an auxiliary linear regression model with patient ID as the dependent variable, and variance inflation factors (VIFs) were calculated. Except for the interaction term and its constituent terms, which showed elevated VIFs (7–16) due to structural multicollinearity, all remaining variables had VIFs below 7 (range 1.0–6.6), well below the conventional threshold of 10. No substantial multicollinearity was detected that would compromise the stability of the treatment effect estimates (Supplementary Table S6). For the LMM, the normality of residuals was assessed using the Shapiro–Wilk test and visual inspection of Q-Q plots. The control group showed a residual *p*-value of 0.650, while the treatment group showed *p* = 0.001, with Q-Q plots indicating that residuals approximately followed the diagonal line and deviations were mainly driven by a few extreme values in the tails (Supplementary Figure S1). Given the robustness of linear mixed models to mild departures from residual normality, the model results were considered reliable.

Response rate was defined as the proportion of patients with a ≥ 50% reduction in seizure frequency. Because all patients were non-responders at month 3, maximum-likelihood GLMM and generalized estimating equation models did not converge. Therefore, between-group comparisons at each follow-up time point were performed using Pearson chi-square tests with Bonferroni correction. The corrected significance level was alpha = 0.0167.

For the IED frequency, the data were skewed and had only two repeated measurements, so the LMM did not converge. We first tried ANCOVA, but the homogeneity of regression slopes assumption was not met (group × baseline interaction, *p* < 0.05). Therefore, we used multivariable linear regression (MLR) with an interaction term. To evaluate the effect of rTMS at different baseline IED frequency levels, patients were divided into low-baseline and high-baseline subgroups according to the median baseline IED frequency. In each subgroup, post-treatment IED frequency was compared between the treatment and control groups using independent-samples t tests or unequal-variance t tests. Effect size was expressed as Cohen’s d. Within-group changes in IED frequency before and after treatment were tested with the Wilcoxon signed-rank test.

Models are specified using Wilkinson notation, where * denotes main effects and their interaction, and (1 | patient_id) denotes a random intercept for each patient. “seizure_frequency” represents the monthly seizure frequency, “pct_reduction” represents the percentage reduction in seizure frequency, “post_IED” represents the post-treatment IED frequency, and “baseline_freq,” “baseline_IED” represents the baseline monthly seizure frequency and baseline IED frequency, respectively. The model formulae are as follows:

1. GLMM: log(seizure_frequency) ~ group * follow_up_time + baseline_freq + age + disease_duration + sex + seizure_type + (1 | patient_id)
2. LMM: pct_reduction ~ group * follow_up_time + baseline_freq + age + disease_duration + sex + seizure_type + (1 | patient_id)
3. MLR: post_IED ~ baseline_IED * group

To control for Type I error inflation due to multiple comparisons, a hierarchical correction strategy was adopted. The primary outcome—pairwise comparisons of seizure frequency at the three follow-up time points from the Poisson GLMM (Supplementary Table S1)—was adjusted using the Sequential Bonferroni method (*α* = 0.05/3 = 0.0167). For secondary outcomes involving multiple comparisons, including within-group before-after comparisons of seizure frequency (six tests, Supplementary Table S2), between-group comparisons of percentage reduction in seizure frequency (three tests, Table 2), and responder rate comparisons (three tests, Table 3), Bonferroni correction was applied with corrected alpha levels of 0.0083, 0.0167, and 0.0167, respectively. Within-group changes in IED frequency (Table 4) and the group × baseline IED interaction in a multivariable linear regression model (Supplementary Table S3) are also included as secondary outcomes but do not involve multiple comparisons. Exploratory analyses (post-treatment IED frequency subgroup analysis, Supplementary Table S4) and descriptive analyses (baseline characteristics, Tables 1 and 5; seizure frequency adjusted estimates, Table 6) were not adjusted for multiple comparisons and are presented as supportive or hypothesis-generating evidence.

**Table 2:** Between-group comparison of the percentage reduction in seizure frequency.

Group	Percentage reduction at month 1 (95% CI)	Percentage reduction at month 2 (95% CI)	Percentage reduction at month 3 (95% CI)
Control	3.9% (−1.6 to 9.4%)	0.6% (−4.9 to 6.1%)	−8.4% (−14.7% to −2.2%)
Treatment	64.6% (58.9 to 70.3%)	73.2% (67.5 to 78.9%)	13.3% (6.9 to 19.7%)
*P*-value (group comparison)	< 0.001	< 0.001	< 0.001

All analyses were adjusted for baseline seizure frequency, age, disease duration, sex, and seizure type. *P*-values were corrected using the Bonferroni method (*α* = 0.05/3 = 0.0167).

**Table 3:** Between-group comparison of responder rates at each follow-up time point.

Visit	Responder in treatment group (*n* = 43)^a^	Responder in control group (*n* = 43)^a^	*χ*^2^ value	*p* value^b^
Follow-up first month	39 (90.7%)	0 (0.0%)	71.362	< 0.001
Follow-up second month	41 (95.3%)	0 (0.0%)	78.356	< 0.001
Follow-up third month^c^	0 (0.0%)	0 (0.0%)	—	—

^a^ Data are presented as *n* (%); ^b^ Pearson’s chi-square test was applied with a Bonferroni-corrected significance level of 0.0167; ^c^ No between-group comparison was conducted at month 3 because no responders were present in either group.

**Table 4:** Within-group comparison of pre and post-treatment IED frequency.

Group	N	visit	Median (IQR)	Z value	*p*-value
Treatment	19	Pre-treatment	11.400 (8.000 to 12.300)	−3.825	< 0.001
		Post-treatment	6.800 (5.700 to 7.600)		
Control	21	Pre-treatment	11.200 (7.900 to 11.850)	−1.641	0.101
		Post-treatment	10.900 (8.050 to 11.750)		

Wilcoxon signed-rank test; two-tailed, *α* = 0.05.

**Table 5:** Patient characteristics of VEEG-subset.

Patient characteristics	Treatment group (*n* = 19)	Control group (*n* = 21)	Statistic	P (two-sided test)
Age, year, median (IQR)^a^	22.6 (14.6,28.6)	21.2 (16.85,25.0)	*Z* = −0.068	0.946
Disease duration, year, median (IQR)^a^	11.2 (6.7,16.7)	10.4 (7.27,14.0)	*Z* = −0.312	0.755
Sex, male/female, *n* (%)^b^	11(57.9)/8(42.1)	12(57.1)/9(42.9)	*χ*^2^ = 0.002	0.962
Baseline seizure frequency, times/month, $\bar{x}$ ±SD^c^	6.23 ± 1.22	6.27 ± 0.87	*t* = 0.129	0.898
Seizure type, *n* (%)^d^				
GTCS	7(36.8)	4(19.0)		
FBTCS	11(57.9)	16(76.2)	—	0.665
FICS/tonic/GTCS	0(0)	1(4.8)		
Absence/FBTCS	1(5.3)	0(0)		

^a^ Mann–Whitney U test; ^b^ Pearson’s chi-square test; ^c^ independent-samples T test; ^d^ Fisher’s exact test. FICS: Focal impaired consciousness seizure; GTCS: generalized tonic–clonic seizures; IQR, interquartile range; SD, standard deviation.

**Table 6:** Adjusted follow-up seizure frequency.

Group	Adjusted seizure frequency at month 1 (95% CI)	Adjusted seizure frequency at month 2 (95% CI)	Adjusted seizure frequency at month 3 (95% CI)
Control	6.03 (5.60 to 6.51)	6.24 (5.77 to 6.76)	6.80 (6.37 to 7.26)
Treatment	2.07 (1.86 to 2.31)	1.52 (1.33 to 1.74)	5.41 (5.05 to 5.81)
*p*-value (between-group comparison)	< 0.001	< 0.001	< 0.001

All analyses were adjusted for baseline seizure frequency, age, disease duration, sex, and seizure type. Between-group comparisons were performed using pairwise contrasts from the same above GLMM, and the resulting *p*-values were adjusted for multiple comparisons with the Sequential Bonferroni procedure.

All tests were two-sided, and all statistical analyses were performed using SPSS.

No sample size calculation was performed before the study. To assess sample size adequacy, a *post hoc* sample size analysis was performed using PASS 15.0. Based on a randomized controlled trial of cerebellar continuous theta-burst stimulation (24), we assumed that the seizure frequency reduction rates in the active stimulation group (μ1) and sham stimulation group (μ2) were 34 and 9%, respectively. The expected between-group difference (δ) was 25%, and the pooled standard deviation (σ) was set at 40%. Using a two-sample Z test for independent means with equal variances, a two-sided alpha = 0.05, power = 0.80, and 1:1 allocation, 41 participants per group were required, or 82 participants in total. In this study, 86 patients completed the trial, meeting this estimated requirement.

## 3 Results

### 3.1 Patient characteristics

Baseline characteristics were summarized in the ITT set (*n* = 87), as shown in Table 1. In the ITT set, the treatment group comprised 22 males and 22 females, with a median age of 22.30 years (interquartile range, IQR, 14.60–28.38), and the control group comprised 25 males and 18 females, with a median age of 20.50 years (IQR, 16.70–25.50). No statistically significant differences were observed between groups in sex, age, disease duration, baseline seizure frequency, seizure type, epilepsy type, etiology, MRI findings, or combined ASMs types (all *p* > 0.05), indicating balanced baseline characteristics in the ITT set. Across the full cohort, etiologies were structural in 23/87 patients (26.4%), genetic in 4/87 (4.6%), immune in 11/87 (12.6%), infectious in 5/87 (5.7%), and unknown in 44/87 (50.6%); no patient was classified as having a metabolic etiology. MRI lesions were present in 50/87 patients (57.5%). An imaging abnormality was not necessarily judged to be the cause of epilepsy, which explains the difference between the MRI-lesional and structural-etiology counts.

Regarding the specific types of antiseizure medications (ASMs) used: among the two-ASMs regimens, levetiracetam + oxcarbazepine was the most common in both groups (11 cases in the treatment group and 10 in the control group). Among the three-ASM regimens, levetiracetam + oxcarbazepine + valproate was the most common in both groups (19 cases in the treatment group and 20 in the control group). None of the patients had previously undergone epilepsy surgery or related neuromodulation procedures.

### 3.2 The effect of rTMS on seizure frequency

The Poisson GLMM revealed a significant group × time interaction for seizure frequency [*F*(2, 236) = 162.65, *p* < 0.001]. At follow-up months 1, 2, and 3, the treatment group had significantly lower seizure frequency than the control group (all adjusted *p* < 0.001). The greatest intergroup difference was observed at month 2, which decreased by month 3 but remained statistically significant (see Supplementary Table S1).

Estimated marginal means for the group × visit interaction (adjusted for baseline frequency, age, disease duration, etc.) revealed that the adjusted seizure frequencies in the treatment group at months 1, 2, and 3 were 2.07, 1.52, and 5.41 per month, respectively, all significantly lower than those in the control group (6.03, 6.24, and 6.80 per month, see Table 6). The rebound in adjusted seizure frequency at month 3 in the treatment group suggests that the optimal duration of efficacy for the treatment parameters and regimen used in this study was approximately 2 months.

Baseline seizure frequency was entered into the model as a covariate to adjust for its influence. The results indicated a significant effect of baseline seizure frequency on follow-up seizure frequency [*F*(1, 236) = 53.06, *p* < 0.001], while no significant effects were observed for the other covariates, including sex, age, and disease duration (all *p* > 0.05).

Within-group comparisons of seizure frequency at each follow-up time point relative to baseline revealed that in the treatment group, seizure frequency at months 1, 2, and 3 was significantly reduced compared with baseline (all *p* < 0.001, *α* = 0.0083 after Bonferroni correction), with median frequencies dropping from 6.33 per month pre-treatment to 2.00, 1.00, and 6.00 per month, respectively. In the control group, no significant differences were observed at any follow-up time point relative to baseline (all *p* > 0.0083; see Supplementary Table S2). Individual-patient seizure frequency trajectories and group-level mean frequency trajectories are shown in Figure 4.

**Figure 4:**
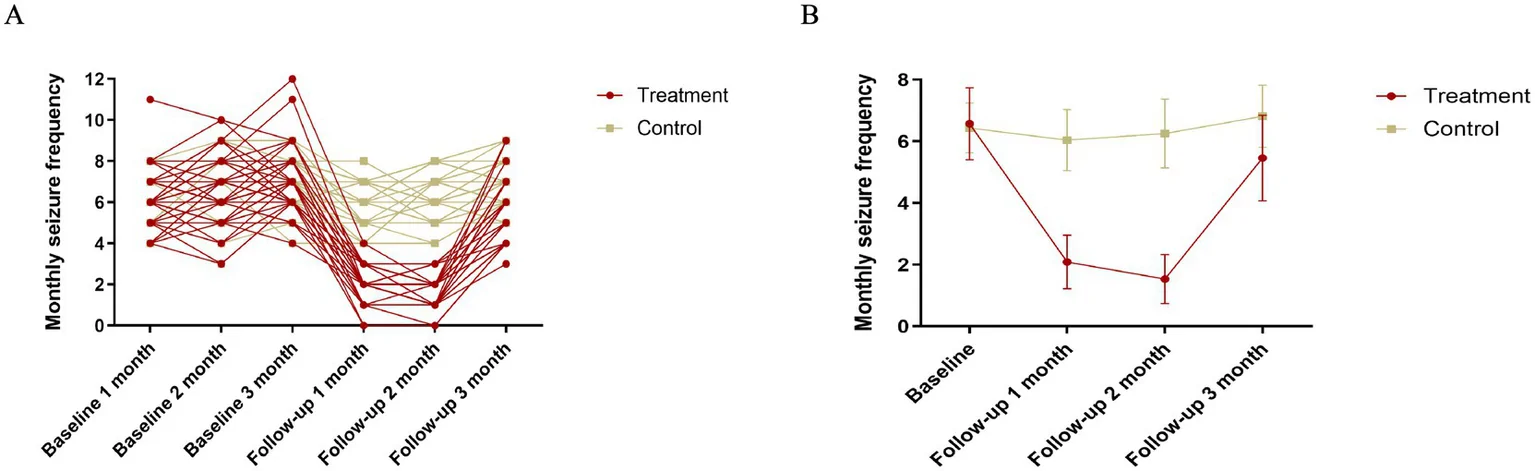
Monthly seizure-frequency trajectories under individual **(A)** and group-level **(B)** across baseline and follow-up. **(A)** Each line represents one patient. The active-rTMS group showed marked reductions at months 1 and 2 followed by rebound at month 3, whereas the sham group remained relatively stable. This figure displays individual raw trajectories for descriptive purposes only; between-group statistical comparisons are presented in Supplementary Tables S1 and Table 6. **(B)** Data are shown as mean ± SD. The active-rTMS group showed the largest reduction at months 1 and 2, followed by partial rebound at month 3.

### 3.3 rTMS effect on seizure frequency reduction percentage

A linear mixed model (LMM) was employed to compare the percentage reduction in seizure frequency between the two groups. A significant group-by-time interaction was observed [*F*(2, 95.85) = 79.60, *p* < 0.001], indicating that the trajectory of percentage reduction over time differed significantly between the treatment and control groups. Estimated marginal means showed that the adjusted percentage reductions in the treatment group at months 1, 2, and 3 were 64.6%, 73.2%, and 13.3%, respectively, all significantly higher than those in the control group (3.9%, 0.6%, and −8.4%; all *p* < 0.001, Bonferroni-corrected; see Table 2 and Figure 5).

**Figure 5:**
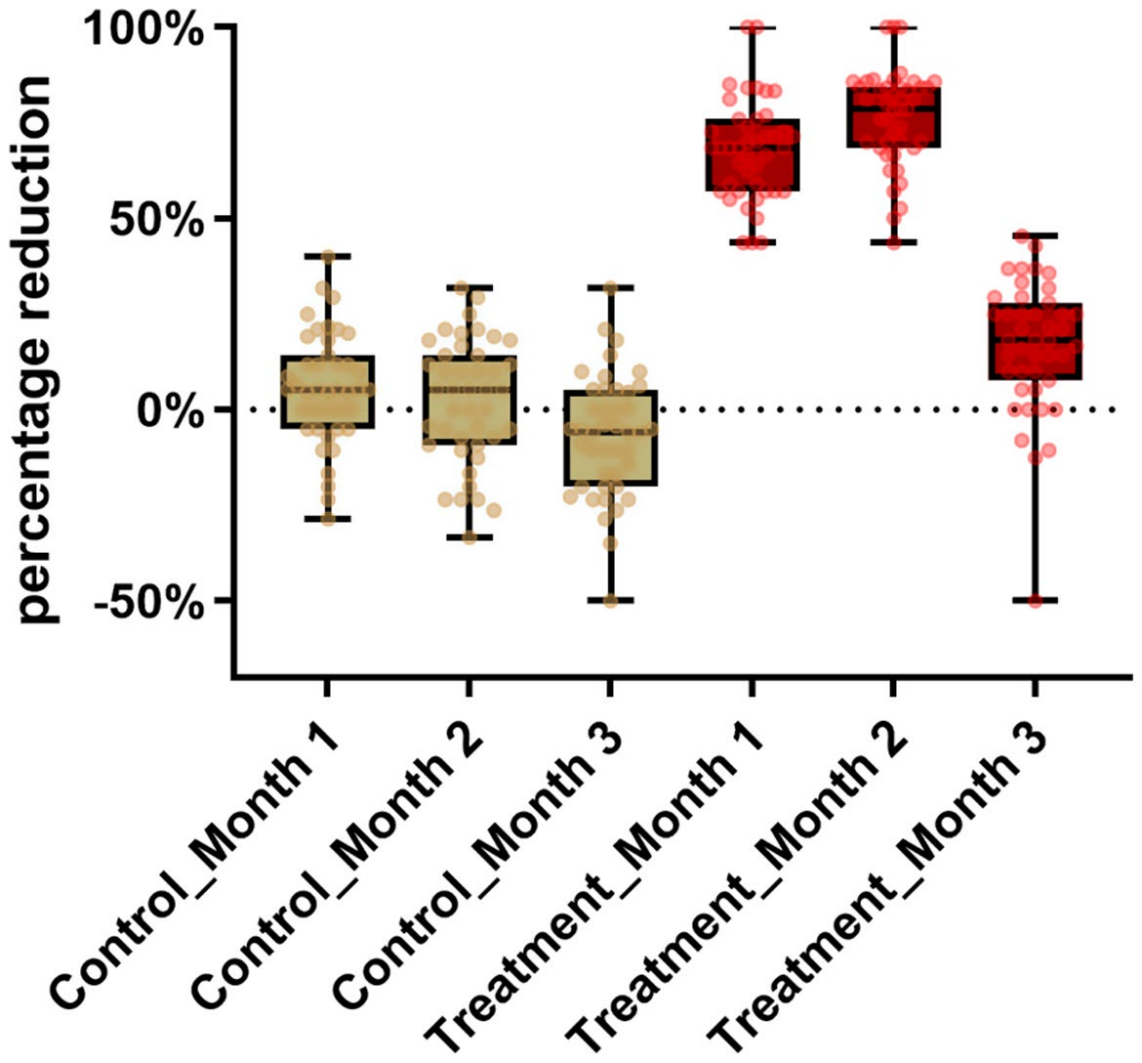
Distribution of seizure-frequency percentage reduction at each follow-up month. The dotted horizontal line denotes 0% reduction; values above this line indicate reduced seizure frequency relative to baseline.

Baseline seizure frequency significantly influenced the percentage reduction [*F*(1, 66.58) = 4.58, *p* = 0.036], while sex, age, and disease duration did not (*p* > 0.05).

### 3.4 rTMS effect on responder rate

The responder rates (proportion of patients achieving a ≥ 50% reduction in seizure frequency) during follow-up were compared between the two groups using Pearson’s chi-square test. In the treatment group, the responder rates at months 1, 2, and 3 were 90.7%, 95.3%, and 0%, respectively, while no responders were observed in the control group at any time point. The between-group differences were statistically significant in the first 2 months (*p* < 0.001). The 0% responder rate at month 3 in the treatment group reflected the magnitude of improvement was below the responder threshold rather than complete disappearance of all residual benefit. In fact, the active rTMS group still showed a lower adjusted seizure frequency and a modest percentage reduction compared with the sham group at this visit. (see Figures 4, 5). These results further suggest that the therapeutic effect of rTMS peaked at month 2 and then declined (see Table 3). The counts of responders and non-responders in each group at each follow-up visit are shown in Figure 6.

**Figure 6:**
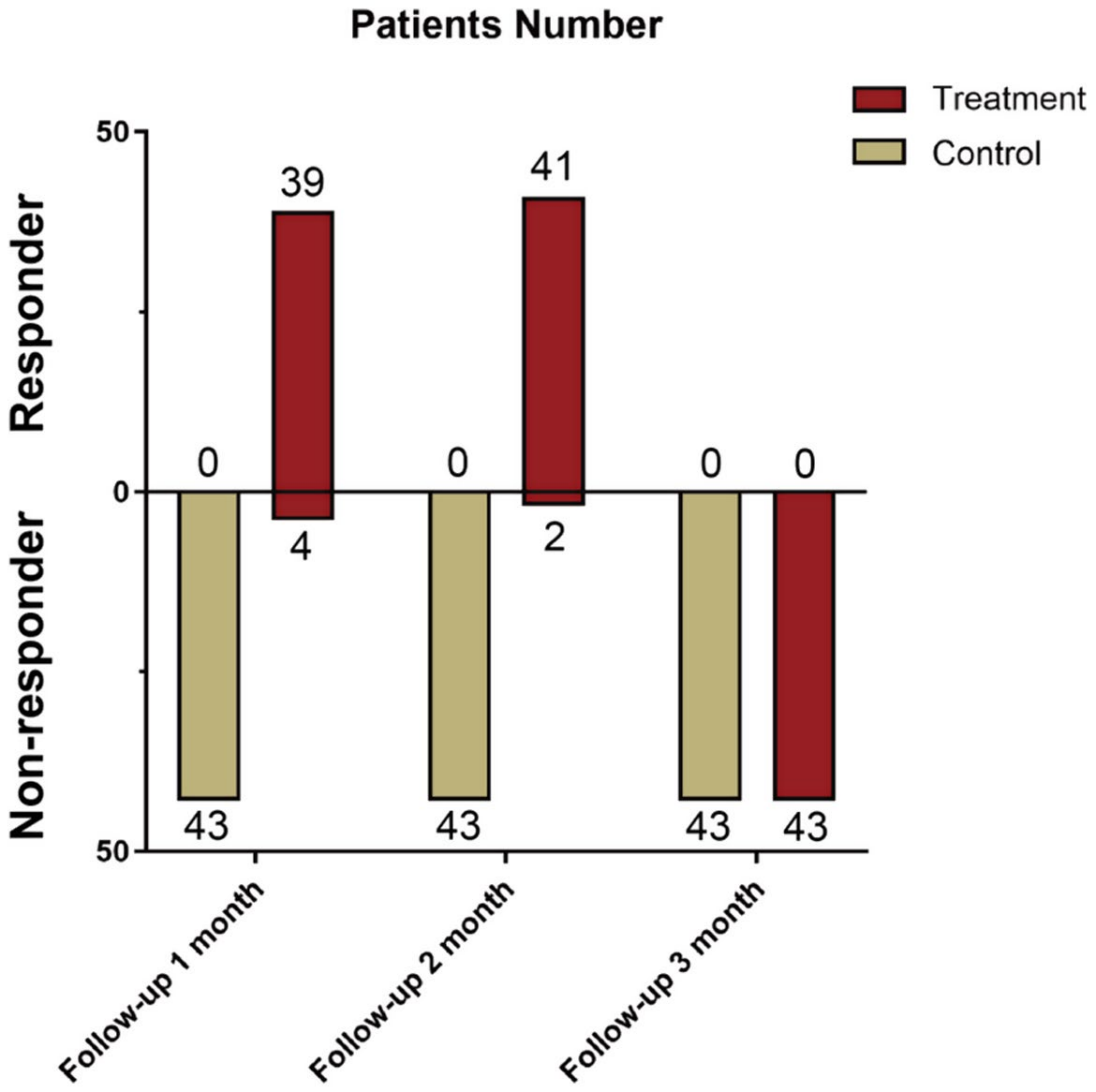
Responder and non-responder counts by group at each visit.

### 3.5 rTMS effect on interictal epileptiform discharges (IEDs)

A subset of 40 patients (19 in the treatment group and 21 in the control group) was randomly selected for VEEG monitoring. Patients’ characteristics in this subset are as follows. The comparison of baseline clinical characteristics between the two groups indicated successful randomization (*p* > 0.05) (see Table 5).

Wilcoxon signed-rank test results demonstrated that IED frequency was significantly lower after treatment than before in the treatment group (*p* < 0.001), indicating a marked reduction following rTMS. In the control group, however, no significant change in IED frequency was observed (*p* = 0.101), indicating that sham stimulation did not significantly affect IED frequency (see Table 4).

Multivariable linear regression revealed a good model fit [*R*^2^ = 0.933, *F* (3,36) = 167.13, *p* < 0.001]. The baseline IED frequency×group interaction term was significant (*p* < 0.001), indicating that the increase in post-treatment IED frequency associated with higher baseline frequency was significantly attenuated in the treatment group compared with the control group. In the control group, a higher baseline IED frequency was strongly associated with a higher post-treatment IED frequency (B = 0.920, *p* < 0.001). In the active TMS group, this association was much weaker (B = 0.536), suggesting that TMS weakened the extent to which baseline IED frequency predicted post-treatment outcomes. This finding suggests that the suppressive effect of rTMS on IED frequency was greater in patients with higher baseline frequency (i.e., those with higher baseline frequency experienced greater relative benefit; see Supplementary Table S3). This finding is visually supported by the regression lines shown in Figure 7.

**Figure 7:**
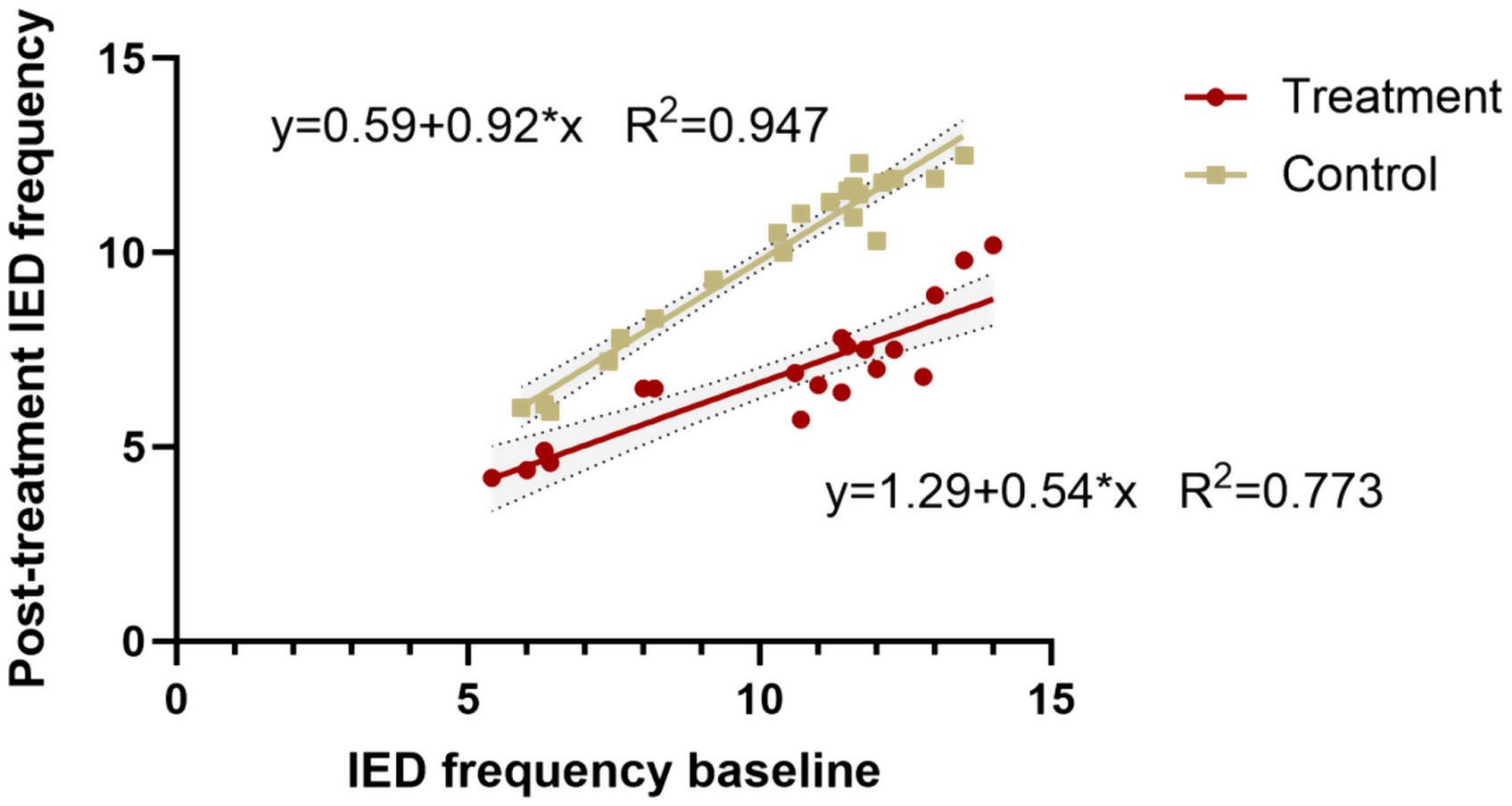
Regression of post-treatment IED frequency on baseline IED frequency by group. Shaded areas are 95% CIs. The slopes differed significantly (interaction B = −0.384, 95% CI − 0.561 to −0.206, *p* < 0.001), indicating a stronger suppressive effect in patients with higher baseline IEDs; post-treatment IEDs increased less steeply with baseline in the active group (slope 0.54) than in controls (slope 0.92).

To confirm the above findings, we further conducted an exploratory analysis of the suppressive effect by baseline seizure frequency. Patients were further divided into low (baseline IED frequency ≤ median) and high-baseline subgroups (> median) based on the median baseline IED frequency. Independent-samples *t*-tests comparing post-treatment IED frequency between the treatment and control groups within each subgroup revealed that, the treatment group had significantly lower IED frequency in both subgroups (low-baseline: *p* = 0.002; high-baseline: *p* < 0.001), indicating an inhibitory effect on IED frequency in both low and high-baseline patients. The effect sizes (high-baseline: *d* = 3.54; low-baseline: *d* = 1.57) suggested a larger effect in high-baseline patients, which was directionally consistent with the interaction term from the multivariable linear regression, which showed that the treatment effect increased with higher baseline frequency (see Supplementary Table S4). However, the very small subgroup sample sizes (8–11 per group) likely resulted in unstable and inflated estimates; hence, this finding should be regarded as exploratory and hypothesis-generating. Confirmatory studies with adequate sample sizes are needed to examine whether baseline IED levels modulate the efficacy of rTMS.

### 3.6 Safety evaluation

All patients voluntarily participated in the study with one lost to follow-up. Adverse events were recorded through seizure diaries and in-person interviews. Four adverse events were reported in the treatment group (muscle twitching, *n* = 1; headache, *n* = 1; dizziness, *n* = 2), and one case of muscle twitching was reported in the sham group (see Supplementary Table S5). There was no significant difference in the incidence of adverse events between the two groups (Fisher’s exact test, *p* = 0.488). All adverse events were mild. No severe adverse events, such as seizures or suicidal ideation/behavior, were reported.

## 4 Discussion

This study examined whether bilateral cerebellar 20-Hz rTMS reduced seizure frequency and IED burden in patients with DRE and characterized the duration and short-term tolerability of any observed effect. The active TMS group showed significantly lower adjusted seizure frequencies at months 1, 2, and 3 (2.07, 1.52, and 5.41/month) compared with controls (6.03, 6.24, and 6.80/month). Adjusted percentage reductions were 64.6%, 73.2%, and 13.3% in the treatment group versus 3.9%, 0.6%, and −8.4% in controls. Responder rates (≥50% reduction) in the treatment group were 90.7%, 95.3%, and 0% at months 1–3, while no responders were observed in the control group. The apparent 0% responder rate at month 3 should be interpreted as loss of responder-level efficacy rather than complete disappearance of all residual benefit. rTMS also significantly suppressed IED frequency, with a greater effect in patients with higher baseline IED frequency. The largest between-group difference occurred at month 2 and diminished but remained significant at month 3, indicating an optimal efficacy duration of approximately 2 months. All adverse events were mild. There was no significant difference in the incidence of adverse events between the two groups (Fisher’s exact test, *p* = 0.488). These findings suggest that high-frequency cerebellar rTMS may offer a non-invasive, tolerable neuromodulatory option for selected patients with drug-resistant epilepsy, but the unusually large early treatment effect should be interpreted cautiously.

In previous studies, the stimulation site was determined based on the international 10–20 EEG electrode system and the location of the lesion. For patients with focal epilepsy, stimulation was targeted at the ictal focus, whereas for those with multifocal or diffuse EEG abnormalities or multiple lesions on MRI, stimulation was applied over the central vertex (Cz in the international 10–20 system) (14, 28, 35–37). However, in patients with multiple, inaccessible, or undefined epileptogenic zones, direct targeting of the epileptic focus is often not feasible. Furthermore, vertex stimulation in DRE lacks therapeutic precision (38). Selection of the stimulation target may therefore consider regions functionally connected to the epileptogenic zone. In cases with lateralized or multiple foci, it is preferable to select a midline region that possesses broad functional connectivity and serves as a common network node (39).

The cerebello-thalamo-cortical network can regulate cortical excitability. Studies have shown that cerebellar stimulation can mediate inhibition of the contralateral primary motor cortex through the Purkinje cell-dentate nucleus-motor thalamus-cortex pathway. This effect is known as cerebellar brain inhibition and can enhance cortical inhibitory output (30–32). Studies have also reported that cerebellar HF-rTMS or iTBS can improve cerebellum-related motor and network function (40–43). Overall, cerebellar HF-rTMS may enhance CBI and induce inhibitory cortical output, thereby reducing epileptiform discharges and clinical seizures.

Evidence on cerebellar TMS for epilepsy remains limited. Brighina et al. first reported that 4 weeks of excitatory cerebellar cortex stimulation (5 Hz, 100 pulses per day) reduced seizure frequency by a mean of 52.9% over the treatment period, but no sustained effect was observed during follow-up (29). In contrast, our study still observed a longer-lasting reduction in seizures after 2 weeks of treatment. The difference in efficacy may be related to stimulation frequency and total pulse number. Wang et al. used inhibitory cTBS targeting the cerebellar dentate nucleus to treat DRE and also found that active stimulation was superior to sham stimulation (28). In their trial, the percentage reduction in seizure frequency was 34% vs. 23% in the active vs. sham groups, and the 50% responder rate was 45% vs. 21%, both showing significant differences. Although the stimulation targets and parameters differed, these findings support the cerebellum cortex Purkinje cell-dentate nucleus-motor thalamus-cortical pathway as a possible network target for DRE.

Most existing TMS studies for epilepsy have used low-frequency stimulation protocols, with target sites including the vertex or individual epileptogenic zones. Several meta-analyses have reported a seizure reduction of approximately 30–34% in drug-resistant epilepsy after low-frequency rTMS intervention (9, 44). Fregni et al. used a short course of 1 Hz rTMS to treat drug-resistant epilepsy associated with cortical dysplasia, and reported seizure frequency reductions of 72%, 53%, and 58% at weeks 2, 4, and 8 after treatment, respectively (37). Sun et al. used 0.5 Hz high-intensity rTMS targeting the epileptogenic zone and reported a seizure reduction of 79.8% during the 8-week follow-up period after a 2-week treatment course (10). In our study, after two 5-day cycles of rTMS, the percentage reductions in seizure frequency at the three follow-up time points over 3 months were 64.6%, 73.2%, and 13.3%, respectively, which are close to the results reported by Sun et al., suggesting that the cerebellum, as an rTMS modulation target, holds promise for the treatment of epilepsy.

In this study, the responder rates during the first 2 months of follow-up reached 90.7 and 95.3%, respectively, but dropped to 0% by the end of the third month, suggesting that the antiepileptic effect of the intervention is markedly time-dependent, peaking at 2 months after intervention and then declining rapidly. This temporal pattern has also been observed in previous low-frequency rTMS clinical trials. In a crossover-controlled study by Melus et al. (45), with a 1-month post-intervention assessment window, the responder rate was 50% and the seizure-freedom rate was 16.7% after five consecutive sessions of 0.5 Hz stimulation; however, the effect persisted for only the first 2 weeks and lost statistical significance by weeks 3–4. In contrast, in the study by Fregni et al. on drug-resistant epilepsy associated with cortical dysplasia, five sessions of 1 Hz rTMS achieved a responder rate of 83.3% in the first 2 weeks after treatment, with 25% of participants experiencing short-term seizure freedom, and the antiepileptic effect was maintained for at least 2 months (37). Sun et al. used 0.5 Hz, 90% rMT high-intensity targeted rTMS, and reported a seizure-freedom rate of 35.5% at the end of a 2-month follow-up after a 2-week treatment course, also achieving a relatively durable clinical response (10). Taken together, there is considerable heterogeneity across studies in both the magnitude of response and the duration of efficacy, which may be related to stimulation parameters, epilepsy etiology, location of the epileptogenic zone, and individual brain plasticity. However, the follow-up duration of existing clinical trials has not exceeded 10 weeks, and evidence for long-term treatment benefit remains lacking (46). Given the rapid decline in efficacy observed in this study, future studies are warranted to explore maintenance stimulation protocols to prolong the treatment benefit.

Regarding safety, TMS-related adverse reactions are usually mild, such as headache or dizziness, and the overall risk of inducing seizures is low (5). It should be noted that a small number of studies reported that HF-rTMS directly targeting the epileptogenic zone may induce clinical seizures (47). In this study, stimulation targeted the cerebellar cortex rather than the epileptogenic zone. All adverse events were mild. No severe adverse events, such as seizures or suicidal ideation/behavior, were reported. Based on previous clinical experience with high-frequency cerebellar stimulation in stroke rehabilitation and spinocerebellar ataxia (48–50), the risk of seizure induction with this protocol appears relatively low.

Currently, the scientific community still holds that the beneficial effects of rTMS on reducing seizures lack robust confirmatory evidence and comparative efficacy data (51). A recent meta-analysis showed that although low-frequency rTMS can significantly reduce seizure frequency in the short term, the overall effect is only small to moderate (46). Some studies have even suggested that rTMS may exacerbate seizures during treatment and lead to a rebound in seizure frequency after an initial reduction (52).

Neuromodulation techniques for epilepsy are not limited to rTMS. As another non-invasive neuromodulation technique, tDCS appears to be relatively more effective for temporal lobe epilepsy, whereas rTMS may be more suitable for epilepsy of neocortical origin (53). In a meta-analysis and systematic review by Chen et al. (53) on cathodal tDCS for drug-resistant epilepsy, tDCS was reported to significantly reduce seizure frequency, with a stronger effect at 2 months than at 1 month; *post hoc* estimates indicated an approximately 28% reduction in seizure frequency at 1 month and approximately 49% at 2 months after treatment. The results of another study were slightly different: a meta-analysis by Hendi et al. (54) showed that after cathodal tDCS, the reduction in seizure frequency at 4 weeks of follow-up was greater than that at 8 weeks (45.39% vs. 39.34%).

Other invasive neuromodulation techniques include VNS (implantable vagus nerve stimulation), RNS (closed-loop responsive neurostimulation), and DBS (deep brain stimulation). A meta-analysis by Touma et al. (55) from the ILAE Surgical Therapies Commission summarized the efficacy of these three implantable neuromodulation approaches. In short-term follow-up of blinded RCTs, seizure frequency reductions for VNS, RNS, and DBS were approximately 24.5–27.9%, 41.5%, and 40.4%, respectively; long-term open-label follow-up showed that the efficacy of RNS and DBS further improved over time, with median seizure reductions of up to 70–75% at 7–9 years of follow-up, whereas long-term evidence for VNS remained relatively limited (56).

This study has several limitations. First, it was a single-center study with a limited sample size, and the trial was not registered before study initiation. Second, only short-term efficacy was assessed, so the effect of repeated treatment, the optimal treatment interval, and long-term safety remain unclear. Larger, longer-term, and more rigorously designed blinded studies are required in the future to establish the long-term efficacy of rTMS. Third, the sham condition was not physiologically or sensorily matched to active stimulation. Rotating the coil by 90 degrees produced different auditory, tactile, vibration, and thermal sensations, and blinding effectiveness was not assessed. This constitutes a substantial risk of performance, expectation, and reporting bias and means that the between-group difference cannot be attributed solely to the physiological effect of rTMS. Future studies should employ a sham stimulation coil to imitate the sound and tactile sensations in active stimulation group and use a questionnaire afterwards to estimate the blinding effectiveness in patients. Fourth, the primary clinical outcome relied on patient/family seizure diaries, which may introduce recall bias, expectation bias, and under or over-reporting of seizures. Caregiver support and diary completeness were not measured systematically, so differential under-reporting among less-supported patients cannot be excluded. Fifth, IED changes were not continuously monitored during follow-up, and cognitive, emotional, and mental status were not systematically assessed. Future studies should include long-term VEEG monitoring and neuropsychological scales during follow-up to clarify the relationship among electrophysiological improvement, seizure reduction, and functional benefit. Sixth, sex data were collected via patient self-report and confirmed with identification documents, but stratified analyses by lesion location, seizure type, or sex were not fully performed. Future work should explore potential sex differences in treatment response according to the SAGER guidelines. Seventh, fixed scalp landmark targeting without neuronavigation or subject-specific electric-field modeling may have introduced substantial variability in the stimulated cerebellar region and delivered dose; target engagement was not verified. In addition, the archived EEG protocol did not retain exact acquisition/filter settings, a prespecified numerical quality threshold, or independent reader scores for inter-rater reliability; therefore, differential artifact contamination cannot be completely excluded. Finally, since Purkinje cell regulation of DCN is not simple on–off inhibition, the rebound period after firing may affect downstream nuclear output (57). Future studies should combine neuronavigation to target different DCN regions based on seizure type and should optimize stimulation parameters for different patients.

In summary, this study suggests that 20 Hz rTMS targeting the cerebellar cortex may reduce seizure frequency and IED frequency in patients with DRE, with good short-term tolerability. Its potential effect may be related to cerebello-thalamo-cortical network modulation and enhanced cortical inhibition. These findings require confirmation in prospectively registered, adequately blinded, multicenter trials with objective long-term outcome monitoring.

## Data Availability

The raw data supporting the conclusions of this article will be made available by the authors, without undue reservation.
