# Supplementary material for: Bilateral cerebellar high-frequency rTMS for refractory epilepsy: a randomized, single-blind, sham-controlled study
Source: Front Neurol. 2026 Oct 29;17:1952421. doi: 10.3389/fneur.2026.1952421 (PMC13754683; doi:10.3389/fneur.2026.1952421)
Supplement: Supplementary file 1 [file Data_Sheet_1.pdf]

Supplemental information

Table S1 Between-group comparison of follow-up seizure frequency

Visit	Difference value (control − treatment)	95%CI	Standard error	Unadjusted p-value	Adjusted p-value
Follow-up first month	3.961	(3.510, 4.413)	0.229	< 1×10-10	< 0.001
Follow-up second month	4.723	(4.231, 5.215)	0.250	< 1×10-10	< 0.001
Follow-up third month	1.388	(0.957, 1.819)	0.219	1.144×10-9	< 0.001

Note: P-values were adjusted for multiple comparisons using the sequential Bonferroni method (α= 0.05/3 = 0.0167).

Table S2 Comparison of pre- and post-treatment seizure frequency among the two groups

Group	Visit	Pre-treatment median (IQR)	Post-treatment median (IQR)	Z value	P value[a]
treatment	Follow-up first month	6.33 (6.00, 7.33)	2.00 (2.00, 3.00)	-5.720	< 0.001
	Follow-up second month	6.33 (6.00, 7.33)	1.00 (1.00, 2.00)	-5.718	< 0.001
	Follow-up third month	6.33 (6.00, 7.33)	6.00 (4.00, 6.00)	-4.729	< 0.001
control	Follow-up first month	6.33 (6.00, 7.00)	6.00 (5.00, 7.00)	-2.548	0.011
	Follow-up second month	6.33 (6.00, 7.00)	6.00 (5.00, 7.00)	-1.169	0.242
	Follow-up third month	6.33 (6.00, 7.00)	7.00 (6.00, 7.00)	-2.522	0.012

a Wilcoxon signed-rank test with Bonferroni-corrected significance level of 0.0083 (= 0.05/6).

Table S3 Multivariable linear regression: predictors of post-treatment IED frequency (with interaction terms)

Variable	B value (95% CI)	Standard error	p value
baseline IED frequency	0.920 (0.789 to 1.052)	0.065	< 0.001
group (treatment)	0.701 (-1.171 to 2.572)	0.923	0.453
group × baseline IED frequency	-0.384 (-0.561 to -0.206)	0.087	< 0.001
constant	0.585 (-0.794 to 1.964)	0.680	0.395

Note: Post-treatment IED frequency served as the dependent variable. $R^2 = 0.933$, adjusted $R^2 = 0.927$, $F(3,36) = 167.13$, $p < 0.001$. B value, unstandardized coefficient.

Table S4 Subgroup analysis of between-group comparison of post-treatment IED frequency

Subgroup	Group	N	Post-treatment IED frequency ($\bar{x} \pm SD$)	T value	Degrees of freedom (df)	P value	Cohen's d
Low-baseline	control	11	8.49 ± 2.05	3.686	15.98	0.002	1.57
	treatment	11	5.86 ± 1.18				
High-baseline	control	10	11.64 ± 0.64	6.934	9.718	< 0.001	3.54
	treatment	8	8.16 ± 1.30				

Note: The t-test was performed under the assumption of unequal variances, as Levene's test was significant for both subgroups (low-baseline: $p = 0.028$; high-baseline: $p = 0.012$).

Table S5 Statistical analysis of adverse events during the study

Adverse effect	Treatment Group (n=43)	Control Group (n=43)	P value[a]
Muscle twitches	1 (2.3%)	1 (2.3%)	0.488
Headache	1 (2.3%)	0 (0.0%)	
Dizziness	2 (4.7%)	0 (0.0%)	
None	39 (90.7%)	42 (97.7%)	

Note: Fisher's exact test

Table S6 The VIF diagnostic results of the linear mixed-effects models

	Specific variable	VIF	Explanation
Core variables	group	7.081	structural multicollinearity (see explanation below)
	follow-up time	10.000	structural multicollinearity (see explanation below)
	group × time interaction	16.000	structural multicollinearity (see explanation below)
Continuous covariates	age	6.578	clinically correlated with disease duration
	disease duration	6.617	clinically correlated with age
	baseline seizure frequency	1.165	no multicollinearity

	Specific variable	VIF	Explanation
Categorical covariates	sex	2.082	no multicollinearity
	seizure-type dummy variables	1.048 ~ 1.659	no multicollinearity

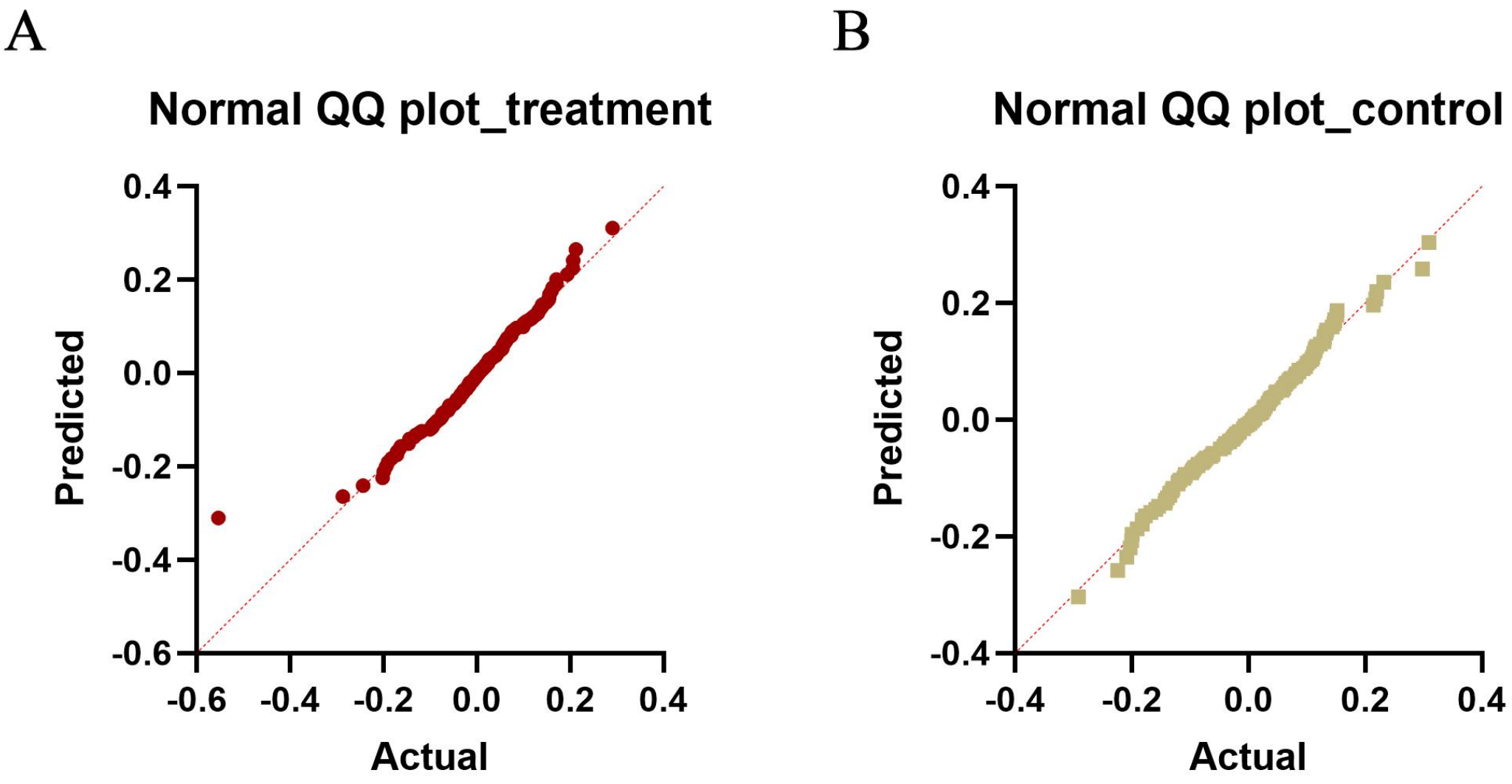

Fig S1. The normal Q-Q plots of the residuals from the linear mixed model
(A) Treatment group; (B) Control group. The Shapiro-Wilk test yielded $p = 0.001$ for the treatment group and $p = 0.650$ for the control group. The Q-Q plot for the treatment group showed that the residuals approximately followed the diagonal line, with deviations mainly driven by a few extreme values in the tails
